# *Aureobasidium pullulans*: a microbiome-based perspective from global biomes to edible plant tissues

**DOI:** 10.3389/fpls.2025.1652366

**Published:** 2025-09-24

**Authors:** Nina Bziuk, Birgit Wassermann, Samuel Bickel, Reza Omidvar, Andrea Manica, Gabriele Berg

**Affiliations:** ^1^ Austrian Centre of Industrial Biotechnology (ACIB), Graz, Austria; ^2^ Institute of Environmental Biotechnology, Graz University of Technology, Graz, Austria; ^3^ SAN Agrow Holding GmbH, Herzogenburg, Austria; ^4^ Microbiome Biotechnology, Leibniz Institute for Agricultural Engeneering and Bioeconomy (ATB), Potsdam, Germany; ^5^ Institute for Biochemistry and Biology, University of Potsdam, Potsdam, Germany

**Keywords:** one health, crop protection, global occurrence, *Aureobasidium pullulans*, edible microbiome

## Abstract

*Aureobasidium pullulans* is a globally distributed fungus commonly found in plant-associated and anthropogenic environments. Known for its antagonistic activity against plant pathogens, it is widely used as a biocontrol agent in sustainable agriculture. Despite its prevalence in edible plant tissues and frequent environmental exposure, its broader role within microbiomes and potential relevance for human health remain underexplored. In this perspective article, we highlight the global distribution of *A. pullulans* based on publicly available sequencing data and examine its ecological function from a microbiome-based viewpoint. Our synthesis supports the view of *A. pullulans* as a safe, plant-beneficial symbiont with high value for sustainable crop protection and potential relevance for the One Health framework. Future microbiome research should further explore its functional roles within plant and human-associated microbiomes to better harness its benefits while ensuring biosafety across ecosystems.

## Introduction

The fungus *Aureobasidium pullulans* (De Bary) Arnaud, commonly known as the ‘black yeast’, was first described 150 years ago (Cooke, 1959). At that time, like the majority of microorganisms, *A. pullulans* was subjected to an anthropocentric perspective on the microbial world, i.e., the mere presence of a microorganism implies disease (Heidenreich et al., 1997). Though ahead of the times, Cooke discussed the ecological life history of *A. pullulans* in 1959 and highlighted that more intense studies may demonstrate its independence from saprobic and pathogenic strains (Cooke, 1959). The discovery of the microbiome, a term first defined by Whipps et al. (1988) and newly conceptualized by Berg et al. (2020) has provided an alternative to the anthropocentric perspective on microbial life. Microorganisms are ubiquitous providers of key ecosystem services and are, thus, intrinsically associated with the health of eukaryotic hosts. This has led to the definition of the holobiont, which refers to a host organism together with all of its associated microorganisms, including bacteria, archaea, fungi, viruses, and protists, forming a complex ecological unit (Vandenkoornhuyse et al., 2015). This concept emphasizes that the biology, evolution, and health of the host cannot be fully understood without considering its microbial interactions and co-evolutionary dynamics. Furthermore, the microbiome interconnects holobionts; for example, plant-associated bacteria in food can withstand human digestion (Wicaksono et al., 2022) and may inhabit the human gut, representing an underexplored but important component of the exposome (Wicaksono et al., 2023a), which is defined as the sum of exposures to which an individual is subjected during their lifespan.

*A. pullulans* is a frequent member of the environmental microbiome. Due to its targeted antagonistic activity, the fungus can protect crops against various plant pathogens, such as *Monilinia laxa*, *Botrytis cinerea, Alternaria alternata, and Fusarium spp* (Zajc et al., 2020; Iqbal et al., 2021; Wachowska et al., 2021; Di Francesco et al., 2023). Initially, *A. pullulans* was categorized into four subspecies: *A. pullulans* var. *pullulans*, var. *melanogenum*, var. *subglaciale*, and var. *namibiae* (Zalar et al., 2008). However, significant genomic differences among these groups warranted their reclassification as four distinct species: *A. pullulans, A. subglaciale, A. namibiae*, and *A. melanogenum* (Gostinčar et al., 2014). This revised taxonomy is particularly important for biotechnological applications in agriculture, as it clearly distinguishes *A. melanogenum* – a species with strains that may possess pathogenic potential for humans – from the agriculturally relevant species *A. pullulans* (Gostinčar et al., 2014; Černoša et al., 2025). *A. pullulans* has been considered safe for various agricultural applications (European Food Safety Authority, 2013; Prasongsuk et al., 2018), and its unparalleled global distribution and use for a sustainable economy (Rensink et al., 2024) calls for studying it in relation to the One Health concept. The importance of *A. pullulans* as an effective biocontrol agent, as well as its applicability in diverse sectors of the sustainable food industry, has been comprehensively reviewed by Di Francesco and colleagues (Di Francesco et al., 2023). However, a microbiome-based perspective on the global and host-associated role of *A. pullulans* is still missing. In this perspective paper, we review the literature on *A. pullulans* occurrence from a microbiome-based perspective to gain new insights into its global prevalence in different biomes and the potential for human exposure by representing a common member of the edible plant microbiome.

## *A. pullulans* is prevalent in anthropogenic environments

*A. pullulans* is known for its host- and non-host-associated lifestyles. The fungus has been detected in numerous ecosystems, ranging from soils (Ignatova et al., 2015; Ademakinwa and Agboola, 2016; Bennamoun et al., 2016), freshwater and marine environments (Gunde-Cimerman et al., 2000; Wang et al., 2009), deserts and drylands (Coleine et al., 2021), glaciers’ ice and permafrost (Branda et al., 2010; Sannino et al., 2020), as well as in the air and atmosphere (Shelton et al., 2002; Griffin, 2007). *A. pullulans* survives in acidic and alkaline surroundings (Cooke, 1959), and saline soils (Bennamoun et al., 2016). Due to its prevalence in extreme habitats, the fungus was described as a polyextremotolerant microorganism (Gostinčar et al., 2023) because it can survive cold as well as hot temperatures up to 50°C (Zajc et al., 2020). However, *A. pullulans* does not grow well at 37°C (Zajc et al., 2020). Interestingly, the fungus does not show substantial specialization in any of these habitats at the genomic level (Gostinčar et al., 2019). Frequent recombination between *A. pullulans* strains could diminish the structuring of the global *A. pullulans* population (Gostinčar et al., 2019).

**Figure 1:**
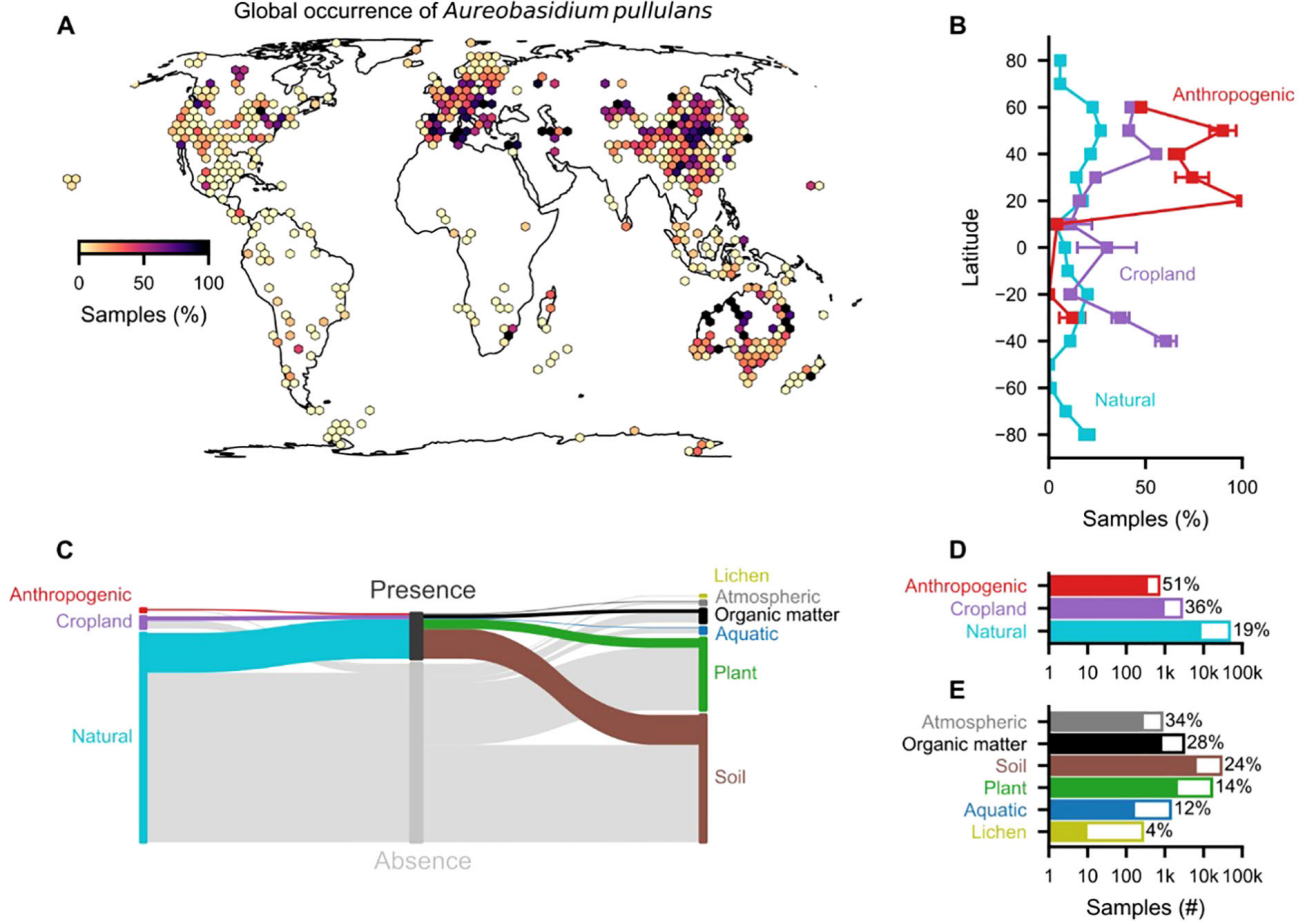
Global occurrence of *A. pullulans*. **(A)** Percentage of samples with *A. pullulans* out of 515 studies based on ITS sequences (ITS1, ITS2, full-length ITS), showing a total of 57’184 samples. **(B)** Latitudinal occurrence patterns in different environments (mean ± SE). **(C)** Distribution of samples with the presence/absence of *A. pullulans* in different environments and sample types. **(D)** Total number of samples in different environments and **(E)** sample types; percentages are represented by the colored part of the bars and indicate the fraction of samples with *A. pullulans*. The 50’084 samples were obtained from the GlobalFungi database (02.11.2023; Větrovský et al., 2020).

We conducted a taxonomy-based search for *A. pullulans* in the GlobalFungi database (Větrovský et al., 2020), which contains high-throughput sequencing metabarcoding studies, to illustrate its global distribution (**Figure 1**). We used two search terms to query the database: (i) an empty prompt to retrieve all samples and (ii) “*Aureobasidium pullulans*” to obtain samples where *A. pullulans* was detected. These two tables were merged, and a total of 57’184 samples were obtained (as of 02.11.2023). We included only samples from 515 studies that were not flagged as “manipulated”, used non-nested primers (covering ITS1, ITS2, or full-length ITS), and contained at least 500 samples per study. This resulted in a total of (i) 50’084 total samples and (ii) 10’191 samples in which *A. pullulans* was detected. Due to the compositional nature of the sequencing data and the high variability among reads between the different primers (**Supplementary Figures 1, 2**), we used presence/absence to delineate the global distribution of *A. pullulans*. Nonetheless, with an increasing number of reads, the detection of *A. pullulans* was more likely, and this was reflected in an increased prevalence (**Supplementary Figure 3**). Hence, we used prevalence for the purpose of delineating the occurrence probability of *A. pullulans* globally.

Our analysis demonstrates that *A. pullulans* can occur in various environments. Although *A. pullulans* occurred on all continents, it was more often detected in anthropogenic environments (51% of n = 707 samples) and croplands (36%, n = 2’713) compared to natural environments (19%, n = 46’664). Interestingly, *A. pullulans* was most prevalent in atmospheric samples, including air and dust. The high prevalence in soil (24%, n = 28’433), organic matter (28%, n = 3043), and atmospheric samples (34%, n = 838) suggests that it is often present in our surroundings. Hence, we expect frequent human exposure to *A. pullulans* across different environments with a low risk for hazard incidents (Prasongsuk et al., 2018).

## *A. pullulans* is an effective biological agent in agriculture

The prevalence of *A. pullulans* in croplands is likely attributable to its broad use as an effective biocontrol agent against bacterial and fungal phytopathogens. Several organic disease control products based on *A. pullulans* are already on the market (e.g., Boni Protect, Blossom Protect, Botector) and are highly promising alternatives to problematic chemicals in viticulture and horticulture, both pre- and postharvest. This is of high importance considering, for instance, the European Green Deal proposing a reduction of the use and risk of pesticides by 50% by 2030.

Over the past decades*, A. pullulans* strains have been applied as single organisms, and in combination with other organisms and chemical peptides (Zajc et al., 2020). Nevertheless, most studies focus on fruit crops, whereas the potential impact of *A. pullulans* on cereals and legumes is less explored. Pre-harvest applications have shown effectiveness against pathogens such as *Erwinia amylovora* in apples (Slack et al., 2019; Zeng et al., 2023), *Diplodia seriata* in grapevines (Pinto et al., 2018), and *Verticillium dahliae* in olive trees (López-Moral et al., 2021, 2022). Post-harvest studies have focused mainly on fruits, demonstrating biocontrol of *Botrytis cinerea* in strawberries, apples, and grapes (Schena et al., 2003; Mari et al., 2012; Iqbal et al., 2022), *Monilinia laxa* in stone fruits (Zhang et al., 2010; Di Francesco et al., 2018), or diverse *Penicillium* species in tropical and non-tropical fruits (Ippolito et al., 2000; Janisiewicz et al., 2000; Zhang et al., 2010, p. 20; Mari et al., 2012; Parafati et al., 2017), just to name some examples. In some cases, it has been used successfully in microbial consortia with *Bacillus subtilis* (Bellamy et al., 2022). In a recent study, *A. pullulans* was transmitted via bees to strawberry flowers, resulting in decreased strawberry post-harvest infections with *B. cinerea* (Iqbal et al., 2022). The potential impact of *A. pullulans* on insects and their microbiome has not been assessed so far (Davis and Landolt, 2013; Hung et al., 2015). Overall, *A. pullulans* is mainly used as a direct antagonist towards phytopathogens, and the main mode of action of *A. pullulans* is referred to as a competition for space and nutrients. However, *A. pullulans* might also have the ability, due to its wide genetic equipment, to induce systemic resistance in plants, which was shown lately by Zeng et al. (2023), demonstrating increased gene expression of pathogenesis-related genes. Further evidence for *A. pullulans’* broad spectrum application potential is given by its postulated function in abiotic stress management of coniferous trees under drought stress (Mannaa et al., 2023), underlining the global potential of *A. pullulans*.

Another important consideration for the use of biocontrol agents is their interaction with the native microbiome in plants and soil, although this aspect has been less explored to date. A recent study showed that the dominance of *A. pullulans* on fruit surfaces resulted in a decreased abundance of naturally occurring phytopathogenic fungi and an increased proportion of bacteria with plant growth-promoting properties (Shi et al., 2022). Since the application and occurrence of *A. pullulans* seems to potentially result in benefits for the plant, we suggest that *A. pullulans* could be considered a fungal soterobiont. This term has recently been postulated to describe microorganisms – artificially applied or native to the plant – that can extend the host plant´s immune system by providing active protection against pathogens, which results in resistant phenotypes (Cernava and Berg, 2022). However, both genetic and functional aspects of *A. pullulans*, the host plant, and other members of the plant microbiota must be considered to build a comprehensive understanding of the dynamics within the holobiont.

## *A. pullulans* is a native member of the plant and the edible microbiome

The native plant microbiome assists the host plant in acquiring nutrients, suppressing pathogens, enhancing stress tolerance, and regulating plant hormones (Sánchez-Cañizares et al., 2017; Berg et al., 2022; Gross, 2022). Thus, plants rely on their associated microbiota and are unlikely to survive without them under natural conditions (Paasch et al., 2023). *A. pullulans* displays a plethora of properties and was observed to natively colonize various plants, including wheat (Wachowska et al., 2020), apple (Abdelfattah et al., 2022), grapevine (Wassermann et al., 2021), olive trees (López-Moral et al., 2021), *Ficus* (Singh and Saini, 2008), wildflowers (Choudhury et al., 2011), and seeds of native alpine plants (Wassermann et al., 2019a). In addition, *A. pullulans* was frequently documented to occur in the edible parts of fresh produce, such as apples (Wassermann et al., 2019b; Abdelfattah et al., 2021; Zhimo et al., 2022), cherries (Schena et al., 2003; Molnárová et al., 2014), peaches (Zhang et al., 2010; Molnárová et al., 2014), citrus (Ferraz et al., 2016), and strawberries (Adikaram et al., 2002), and a large body of literature observed *A. pullulans* in berries of grapevine (Fleet, 2003; Martini et al., 2009; Verginer et al., 2010; Grube et al., 2011; Barata et al., 2012; Pinto et al., 2014). However, these observations are mainly based on PCR-based marker gene profiling or microbial cultivation methods, which do not provide direct information regarding actual microbial loads.

Based on the global data set, we found a wide range of sequence reads of *A. pullulans* across the different samples, reaching high percentages in certain samples (**Supplementary Figure 2**). However, microbiome-based quantitative data on the fungal plant microbiota remain limited. To estimate the load of *A. pullulans* in plant tissues, we analyzed a previously published dataset on the apple microbiome (Abdelfattah et al., 2022). The data includes marker gene sequences (ITS) and quantitative real-time PCR (ITS gene copy numbers (GCN) per centimeter of shoot length) measurements for the endophytic microbiota of 61 apple accessions from 11 *Malus* species. The total fungal load ranged from 10^6^ GCN cm^-1^ to 10^9^ GCN cm^-1^ in domesticated apples (Abdelfattah et al., 2022), and *A. pullulans* sequences accounted for 36% to 51% of all fungal GCN in these samples, indicating that the fungus is a native and significant member of the apple microbiome. This example supports *A. pullulans’* environmental prevalence and shows its potential for host interaction, as microbial abundance is a key determinant of ecological relevance and functional impact on the host (Lloréns-Rico et al., 2021).

In general, while all niche-specific microbiota play a functional role for the plant (Trivedi et al., 2020), and for humans as consumers, microbes associated with the edible parts of a plant can pose health benefits and risks (Berg et al., 2015; Kim et al., 2020). The risks are deeply studied, yet human pathogens causing food-borne outbreaks, as well as opportunistic pathogens that cause healthcare-associated infections (Mehta et al., 2017), are still of global concern, accelerated by the drivers of the Anthropocene (Flandroy et al., 2018). However, edible plants are colonized by a huge diversity of microorganisms, and only a very small fraction may have adverse impacts on healthy humans (Berg et al., 2015). Those microbes represent the edible plant microbiome and are an important component of the exposome (Berg et al., 2015; Wicaksono et al., 2023a). Studies suggest that the edible microbiome may positively impact the gut microbiome and human health. Fruit and vegetable-derived bacteria are, despite their low abundance, consistently present in the human gut, enriching the functional diversity of the gut microbiota due to the presence of genes associated with benefits for human health (Wicaksono et al., 2023b). Besides bacteria, eukaryotic organisms are important components of the gut microbiome (Zhang et al., 2022); yet, to our knowledge, no comparable studies on fruit- and vegetable-transmitted fungi in the human gut have been conducted so far (Laforest-Lapointe and Arrieta, 2018). Nonetheless, *A. pullulans* has been detected in stool samples of healthy humans (Maas et al., 2023). In addition, it is known that fungal β-glucans play an important role in the human immune system (Zhang et al., 2022) and also the compounds produced by *A. pullulans* may have potential health benefits for humans (Ikewaki et al., 2023; Raghavan et al., 2023). Analyzing the diversity of fungi and other eukaryotes in the human gut and whether they are delivered via plant consumption will help uncover those microorganisms’ roles for host health.

## Conclusion

*A. pullulans* is a globally distributed fungus commonly found in anthropogenic environments and croplands. Research indicates that *A. pullulans* pose minimal health risks to humans (Gostinčar et al., 2014). Yet, given its widespread occurrence in food and the environment, animal and human exposure is probable, necessitating comprehensive risk assessments to ensure safety. The fungus’s beneficial properties for plants, including pathogen suppression and crop protection, along with its native abundance in wild plants and crops, underscore its potential relevance for future agricultural practices aligned with the One Health framework. Future research should focus on its interaction with the native plant and the environmental microbiome. Further, its role within the human exposome and gut microbiome needs to be explored to better understand its interactions and any potential health implications.
