# Supplementary material for: Aureobasidium pullulans: a microbiome-based perspective from global biomes to edible plant tissues
Source: Front Plant Sci. 2025 Sep 24;16:1652366. doi: 10.3389/fpls.2025.1652366 (PMC12505387; doi:10.3389/fpls.2025.1652366)
Supplement: Supplementary file 1 [file DataSheet1.docx]

**Supplementary Figures**


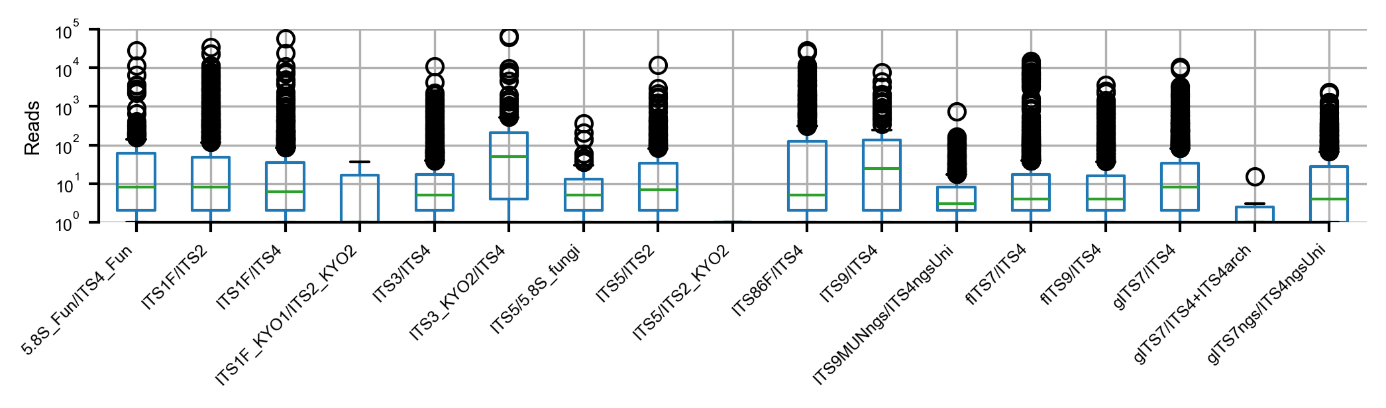


Supplementary Figure 1: Reads associated with A. pullulans for the different primers in the GlobalFungi database.


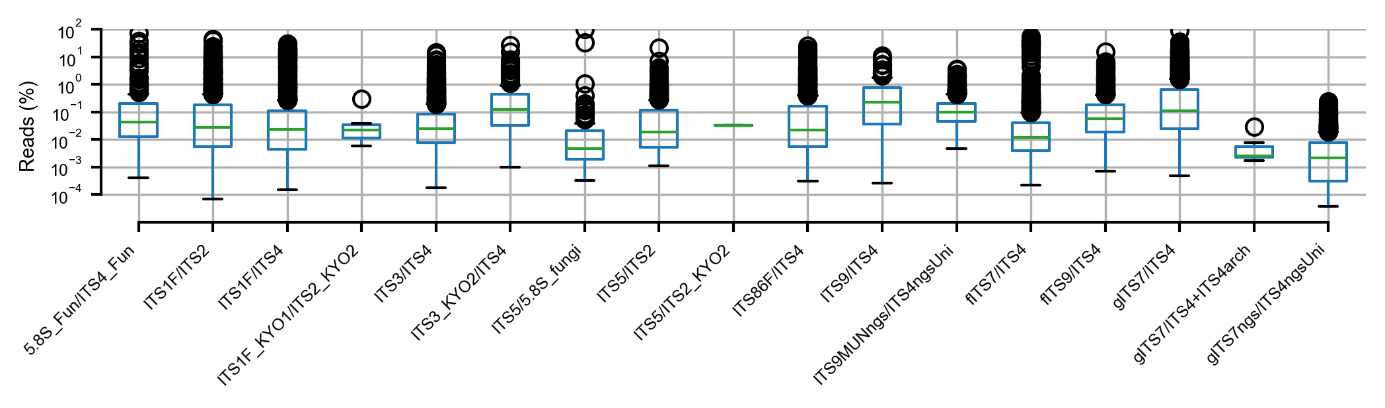


Supplementary Figure 2: Percentage of reads associated with A. pullulans for the different primers in the GlobalFungi database.


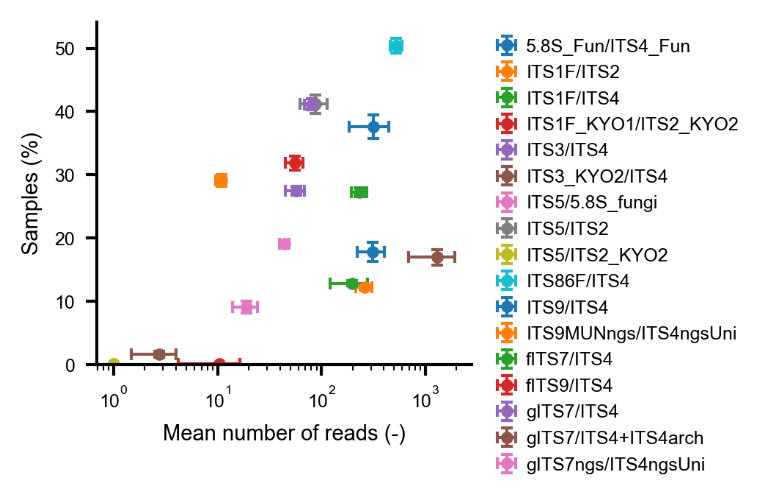


Supplementary Figure 3: Average number of reads of A. pullulans and the percentage of samples it was detected for the different primers. The mean and standard error are shown as markers and whiskers, respectively.
